# Performance Optimization of FD-SOI Hall Sensors Via 3D TCAD Simulations

**DOI:** 10.3390/s20102751

**Published:** 2020-05-12

**Authors:** Linjie Fan, Jinshun Bi, Kai Xi, Sandip Majumdar, Bo Li

**Affiliations:** 1 Institute of Microelectronics, Chinese Academy of Sciences, Beijing 100029, China; fanlinjie19@mails.ucas.ac.cn (L.F.); xikai@ime.ac.cn (K.X.); libo3@ime.ac.cn (B.L.); 2 School of Microelectronics, University of Chinese Academy of Sciences, Beijing 100049, China; 3 Department of Physics, Serampore Girls’ College, Hooghly 712201, India; sandipiitkgp13@gmail.com

**Keywords:** Hall sensors, FD-SOI CMOS, optimization design, 3D simulations, design rules

## Abstract

This work investigates the behavior of fully depleted silicon-on-insulator (FD-SOI) Hall sensors with an emphasis on their physical parameters, namely the aspect ratio, doping concentration, and thicknesses. Via 3D-technology computer aided design (TCAD) simulations with a galvanomagnetic transport model, the performances of the Hall voltage, sensitivity, efficiency, offset voltage, and temperature characteristics are evaluated. The optimal structure of the sensor in the simulation has a sensitivity of 86.5 mV/T and an efficiency of 218.9 V/WT at the bias voltage of 5 V. In addition, the effects of bias, such as the gate voltage and substrate voltage, on performance are also simulated and analyzed. Optimal structure and bias design rules are proposed, as are some adjustable trade-offs that can be chosen by designers to meet their own Hall sensor requirements.

## 1. Introduction

Hall sensors are currently the most widely used magnetic sensors [1]. Compared with other magnetic sensors, Hall sensors are characterized by non-contact, strong anti-interference, and high linearity [2], and they are therefore used in current detection, angle measurement, and geomagnetic field detection. Since the 1940s, with the vigorous development of semiconductor technology, III-V semiconductor materials with low carrier concentrations and high mobility have become the mainstream materials for the fabrication of Hall cells, compared to silicon-based the Hall cells [3]. However, with the fast growth and maturity of integrated circuit technology, Hall integrated circuits with Hall cells and signal processing circuits have emerged, and the properties of III-V semiconductor materials are significantly less attractive for the integration of Hall sensors than are the properties of silicon materials [4].

Therefore, to achieve good performance, it is useful to have a simple set of rules for the customization of sensor designs that are compatible with silicon complementary metal-oxide-semiconductor (CMOS) processes. Compared to the conventional bulk CMOS-based Hall sensors, fully depleted silicon-on-insulator (FD-SOI) technology offers several substantial advantages. The FD-SOI structure not only has the benefits of low voltage and power consumption, strong radiation resistance, a small leakage current, and high integration density [5,6,7], but is also suitable for the integration of Hall sensors due to its smaller film thickness and lower doping concentration.

Several existing studies have investigated the temperature and power performances of SOI Hall sensors via experimental methods [8,9]. Some research has also adopted three-dimensional (3D) physical models to simulate the design optimization of SOI Hall cells [10,11,12,13]. Dolgyi et al. [10] studied the dependencies of electric characteristics on the process parameters of SOI Hall sensors. However, this research was limited to the analysis of electrical characteristics, such as current-voltage characteristics, the threshold voltage, and the breakdown voltage, and presented less discussion on the effects of bias, geometries, or other design parameters on sensitivity, offset voltage, and power consumption. Paun et al. [13] analyzed the behavior of an optimum Hall cell in a partially depleted SOI (PD-SOI) fabrication process by constructing a 3D physical model of the structure and performing simulations; however, the SOI Hall sensor structure in this work did not use the FD state, and an analysis of gate and substrate voltage regulation was absent.

The present work investigates the behavior of FD-SOI Hall sensors with the use of 3D technology computer aided design (TCAD) tools. It focuses on the impacts of design parameters (such as the aspect ratio, thickness, and contact length) and applied bias in the performance of Hall sensors, which includes its sensitivity, offset voltage, power consumption, and temperature characteristics. The remainder of this paper is organized as follows. Section 2 introduces the figures of merits for Hall sensor performance evaluation, the Hall sensor structure, and the TCAD simulation methodology. Section 3 explores the key parameters that can optimize the performance of an FD-SOI Hall sensor via physical simulations. Finally, Section 4 discusses design rules and summarizes the optimum parameter conditions.

## 2. Methodology and Physical Model

### 2.1. Theory

This section briefly summarizes the figure of merit definitions for Hall sensor evaluation. When a current flow orthogonal to an external magnetic field passes through a semiconductor, the carriers are deflected, which results in a voltage difference called the Hall voltage *V_H_* that can be obtained by the following equation:

(1) $${\;V}_{H}=G\;\frac{r_{H}}{nqt}I_{bias}B,$$

where *G* is the geometric correction factor, *r_H_* is the scattering factor of silicon and is usually 1.15, *n* is the carrier density, *q* is the electron charge, *t* is the thickness of the active region, *I_bias_* is the bias current, and *B* is the magnetic field induction [14].

One of the most important parameters of Hall sensors is their sensitivity, which characterizes the sensitivity of the sensor to magnetic field signals. The absolute sensitivity *S_A_* of a Hall sensor can be expressed as follows [1]:

(2) $$S_{A\;}=\frac{V_{H}}{B}=G\;\frac{r_{H}}{nqt}I_{bias}.$$

*S_A_* is not only related to the properties of the material, but is also proportional to the voltage or excitation current. If the influence of the power supply on the sensitivity is eliminated, the current-related (*S_I_*) and voltage-related (*S_V_*) sensitivities of the Hall sensor can be obtained [1]:

(3) $${\;S}_{I\;}=\frac{S_{A}}{I_{bias}}=G\;\frac{r_{H}}{nqt},$$

(4) $${\;S}_{V\;}=\frac{S_{A}}{V_{bias}}{=G\;r}_{H}\;\mu \;\frac{W}{L},\;$$

where *V_bias_* is the bias voltage, *W* and *L* are respectively the width and length of the Hall sensor, and *μ* is the carrier mobility.

At present, one could notice at this point that if a high-sensitivity Hall sensor is to be obtained, its required characteristics include a low carrier concentration, a small active film thickness, and a high aspect ratio. Therefore, the FD-SOI structure, which has a smaller film thickness and a lower doping concentration in the active region, meets the requirements needed to improve the sensitivity of the Hall sensor.

### 2.2. Device

**Figure 1:**
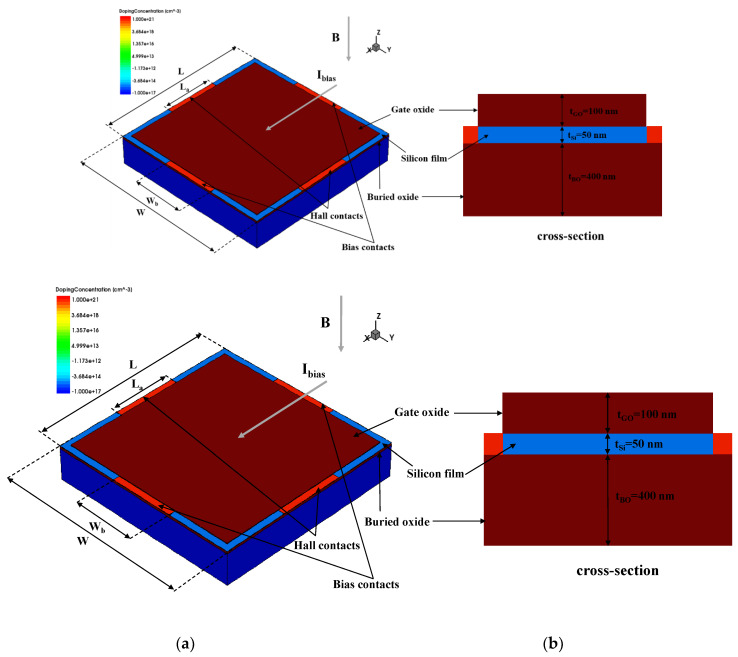
(**a**) 3D geometric model and (**b**) cross-section of the FD-SOI Hall sensor.

In this work, the structure of the Hall sensor was considered symmetrical and orthogonal, and Figure 1 illustrates the 3D geometric model and cross-section of the FD-SOI Hall sensor. The structure consists of a P-type lightly doped silicon film (*N_A_* = 1 × 10^16^ cm^−3^) and four N-type heavily doped contacts (*N_D_* = 1 × 10^21^ cm^−3^) along four sides. The top and bottom layers of the silicon film are gate oxide and buried oxide, respectively. The length (*L*) and width (*W*) of the Hall sensor device range from 15 µm to 45 µm, which are typical values. The length of the Hall contacts (*L_a_*) ranges from 0.25 × *L* to 0.93 × *L* and the width of the bias contacts (*W_b_*) ranges from 0.25 × *W* to 0.93 × *W*, while the four contacts are located at the centers of the four sides of the sensor. The thickness of the silicon film is 50 nm, so that the device can reach the FD state under the corresponding low-doping conditions; this is further discussed in Section 3.3.

The Hall sensor is essentially based on a large FD-SOI metal-oxide-semiconductor field-effect transistor (MOSFET), and the bias contacts are equivalent to the source and drain regions of the conventional MOSFET. An appropriate voltage is applied to the bias contacts, which means that a current path is generated between the source and drain. Therefore, the Hall voltage can be detected at the Hall contacts under the influence of the magnetic induction intensity *B*. A 5 μm thick silicon substrate is used below the buried oxide layer, which is sufficient for the TCAD simulation. By changing the gate (above the gate oxide layer) voltage *V_g_* and the substrate voltage *V_sub_*, the working state of the top silicon film can be adjusted to achieve optimum characteristics.

### 2.3. Numerical Model

For semiconductor devices, the classic carrier transport model [15,16,17] is based on continuity equations. In addition, the following partial differential equation should also be considered to acquire a complete description of semiconductor physical behavior:

(5) $$∆\vec{V}=-\frac{q}{\varepsilon }{\;(p\;-\;n+N}_{D\;}{-N}_{A}),\;$$

where $\vec{V}\;$ denotes the electrostatic potential, *ε* is the material electric permittivity, *q* is the electron charge, *n* is particle density for electrons, *p* is particle density for holes, *N_D_* is the concentration of ionized donors, and *N_A_* is the concentration of ionized acceptors. The solution of the Poisson’s equation in (5) is the electrostatic potential *V*.

Via the use of the Synopsys Sentaurus Device^®^ tool [18], a 3D simulation of Hall sensors was performed. Device simulations can be viewed as virtual measurements of the electrical behavior of a semiconductor device. The device is represented as a meshed finite-element structure. Each node of the device has properties associated with it, such as material type and doping concentration. For each node, the carrier concentration, current densities, electric field, generation and recombination rates, and so on are solved. Specifically, the tool can solve the Poisson’s equation, as well as both electrons and holes continuity equations. The 3D numerical simulation method is employed to study the carrier transport process, namely the electrostatic potential and current distribution, of the Hall sensor in a magnetic field. Basically, at each point of the grid, the three unknowns *V*, *n*, and *p* will be considered first. Furthermore, three equations and corresponding boundary conditions are required to solve the nonlinear systems of partial differential equations. To obtain the correct solution, it is necessary to discretize the three equations and adopt a coupling method, i.e., a generalization of the Newton method, to calculate the proposed initial system via numerical iteration.

The magnetic field acting on the semiconductor Hall sensor was managed by the galvanic transport model. To consider the magnetic field-dependent terms introduced by the effect of the Lorentz force on the carriers, the usual drift-diffusion model including the carrier densities $\vec{J_{n}}$ and $\vec{J_{p}}$ must be rewritten. Within the galvanic transport model, Sentaurus includes the effect of a magnetic field on semiconductors, and the following equation for holes and electrons governs its behavior:

(6) $$\vec{J_{\alpha }}{=\;\mu }_{\alpha }\vec{g_{\alpha }}{\;+\;\mu }_{\alpha }\;\frac{1}{{1\;+\;(\;\mu }_{\alpha }^{*}{B)}^{2}}\;{[\mu }_{\alpha }^{*}\vec{B\;}\times \vec{g_{\alpha }}\;+\mu_{\alpha }^{*}\vec{B\;}{\times \;(\mu }_{\alpha }^{*}\vec{B\;}\times \;\vec{g_{\alpha }})]\;,$$

where *α = n* or *p,* $\vec{g_{\alpha }}$ is the current vector without mobility, $\mu_{\alpha }^{*}$ is the Hall mobility, $\vec{B}$ is the magnetic field vector and *B* is the magnitude of the vector $\vec{B}$ [19]. In addition to the transport equation and Lorentz force, the simulation also takes into account mobility degradation due to rough surface scattering, high doping concentration, and high field saturation. It has been proven that the Synopsys Sentaurus Device^®^ is a TCAD tool capable of designing and optimizing Hall sensors [20,21].

## 3. Experimental Results and Discussion

In this section, with the support of TCAD simulation, the effects of the changes in the structural parameters and the applied bias on the performance of the FD-SOI Hall sensor are evaluated. The design rules are then defined based on the simulation results.

### 3.1. I-V Characteristics

As can be seen from (2) in Section 2.1, the value of *S_A_* of the Hall sensor is related to the applied bias. In addition, the FD-SOI Hall sensor has the basic properties of a conventional MOSFET. Therefore, first, we analyzed the output and transfer characteristics of the FD-SOI Hall sensor without an applied magnetic field.

**Figure 2:**
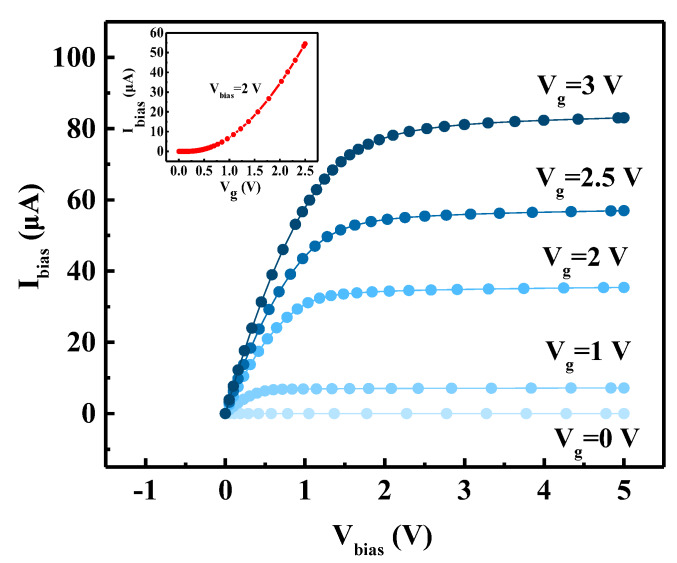
Dependencies of bias current *I_bias_* on the bias voltage *V_bias_* at various gate voltages *V_g_* of the FD-SOI Hall sensor. The inset is the transfer characteristic curve, which shows the variation of *I_bias_* with *V_g_*.

As presented in Figure 2, with the increase of *V_bias_*, *I_bias_* was found to gradually increase and tend to saturation. This is in line with the characteristics of the output curve of the MOSFET, and its formulas in the linear region and saturation region are respectively expressed as follows:

(7) $$I_{bias}=\frac{W}{L}C_{OX}{\mu \;(V}_{g}{-V}_{t}-\frac{m}{2}V_{bias}{)\;V}_{bias},\;$$

(8) $$I_{bias\_sat}=\frac{W}{2mL}C_{OX}{\mu \;(V}_{g}-V_{t}{)}^{2}\;,$$

where *C_OX_* is the gate oxide capacitance per unit area, *V_t_* is the threshold voltage, *m* is the bulk-charge factor, and *I_bias_sat_* is the saturation bias current. Moreover, as presented in the inset of the figure, *I_bias_* increases with *V_g_*; when *V_bias_* is 5 V, *I_bias_* increases from 0.22 nA at *V_g_* = 0 V to 83.04 μA at *V_g_* = 5 V. This means that *I_bias_* can be adjusted by changing the gate voltage, thereby changing the sensitivity; this is further discussed in Section 3.7. In the subsequent sections, unless otherwise specified, the bias added to the device was *V_bias_* = 5 V, *V_g_* = 2.5 V, and *B* = [0, 1] T, which caused the corresponding *I_bias_* to be 56 μA.

### 3.2. Offset Voltage

The offset voltage *V_offset_* characterizes the value of *V_H_* of the Hall sensor at zero magnetic field. Ideally, the *V_offset_* value of the Hall sensor should be equal to zero. However, in practical applications, due to the heterogeneity of the channel material and the asymmetry of the geometric dimensions, *V_offset_* always exists. The *V_offset_* value of traditional Hall sensors currently on the market is typically 10 mV (with *V_bias_* = 3 V) [22]. In the integrated circuit, *V_H_* and *V_offset_* in the Hall sensor are amplified by the subsequent amplifier circuit together; this will lead the signal to saturate, thereby causing the failure to detect the external magnetic field signal [23].

**Figure 3:**
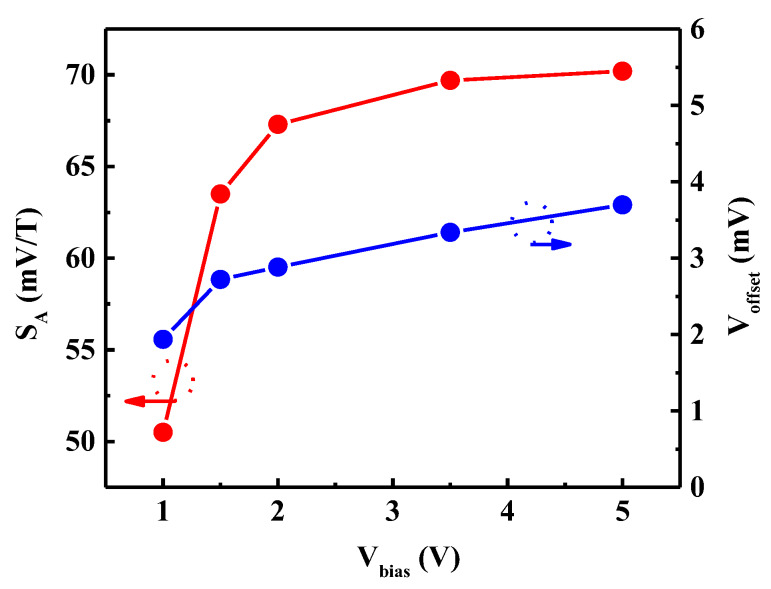
Diagram of absolute sensitivity *S_A_* and offset voltage *V_offset_* of the FD-SOI Hall sensor under different voltage biases *V_bias_*. *V_bias_* defines the sensitivity/offset voltage trade-off that must be properly adjusted based on the application constraints.

Figure 3 presents the changes of *S_A_* and *V_offset_* with the bias voltage. The increase of *V_bias_* leads to the increase of *I_bias_* (Figure 2), which increases *S_A_*. Unfortunately, an increase in bias also causes an increase in *V_offset_*, which ranges from 1.94 mV at *V_bias_* = 1 V to 3.7 mV at *V_bias_* = 5 V. Therefore, *V_bias_* defines the sensitivity/offset voltage trade-off that must be properly adjusted based on the application constraints.

### 3.3. Fully Depleted Structure

The basis for classifying SOI devices as FD is mainly determined by the doping concentration *N_A_* and the thickness of the top silicon film *t_si_*. If the thickness of the silicon film is less than twice the maximum depletion layer width *X_dmax_* under the gate, the device is fully depleted. The value of *X_dmax_* can be determined as follows:

(9) $${\;X}_{dmax}=\sqrt{\frac{{\;4\varepsilon }_{si}\varphi_{F}}{{qN}_{A}}},$$

where *ε_si_* is the dielectric constant of silicon, *φ_F_* is the Fermi potential, *N_A_* is the doping concentration of the silicon film, and *q* is the charge of the electron. By calculation, it was determined that the doping concentration (*N_A_* = 1 × 10^16^ cm^−3^) and thickness (*t_si_* = 50 nm) of the silicon film of the simulated structure meet the conditions of FD.

**Figure 4:**
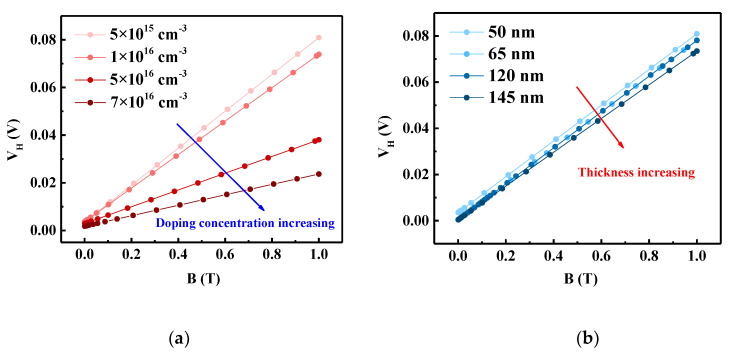
Hall voltage (*V*_H_) versus magnetic induction (*B*) simulated for sensors with different (**a**) doping concentrations and (**b**) silicon film thicknesses.

Figure 4a,b respectively present the simulated *V_H_* versus *B* values for sensors with different doping concentrations and silicon film thicknesses. It is evident that as the doping concentration or silicon film thickness increases, the FD-SOI Hall sensor is no longer in an FD state. Therefore, the value of *V_H_* and the sensitivity of the sensor under the same magnetic field gradually decrease.

### 3.4. Gate-Channel Work Function Difference and Gate Oxide Thickness 

**Figure 5:**
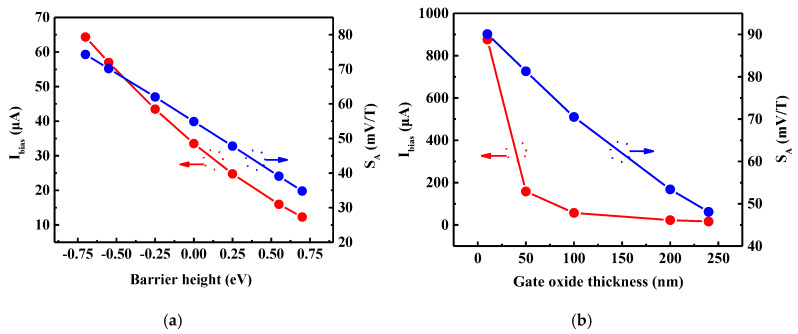
Bias current (*I_bias_*) and absolute sensitivity (*S_A_*) versus (**a**) the barrier height of the gate-channel and (**b**) the gate oxide thickness.

In the sensor structure, when different metals are selected as the gate electrode, the barrier height of the gate metal and silicon film will be different due to the differences in the work functions of the metals. For sensors of the same size, differences in barrier heights directly affect the built-in electric field in the silicon film, resulting in a change in the threshold voltage that in turn affects the value of *I_bias_* at the same value of *V_bias_*. It is clear from Figure 5a that with the increase in the barrier height from −7 eV to 7 eV, the value of *I_bias_* decreases from 64.33 μA to 12.28 μA, a decrease of 80.91%. Additionally, the value of *S_A_* decreases from 74.3 mV/T to 34.8 mV/T, a decrease of 53.16%. Therefore, for the better performance, a metal gate with a small work function should be selected from Hall sensor’s perspective.

The built-in electric field in silicon film is affected not only by the barrier height, but also by the gate oxide thickness. With the gradual reduction of the gate oxide thickness, the more the gate can control the channel formed in the silicon film; therefore, the value of *I_bias_* is greater. As can be seen from Figure 5b, with the increase of the gate oxide thickness from 10 nm to 100 nm, the change of *I_bias_* is about 1 order of magnitude, but the value of *S_A_* only decreases by about 20 mV/T. Via calculation, this is found to result in the *S_I_* value of 10 V/AT at a 10 nm gate oxide thickness being much less than the value of 1237 V/AT at a 100 nm gate oxide thickness. Hence, although the value of *S_A_* increases slightly with the decrease of the gate oxide thickness, the value of *S_I_* decreases substantially.

### 3.5. Aspect Ratios

**Figure 6:**
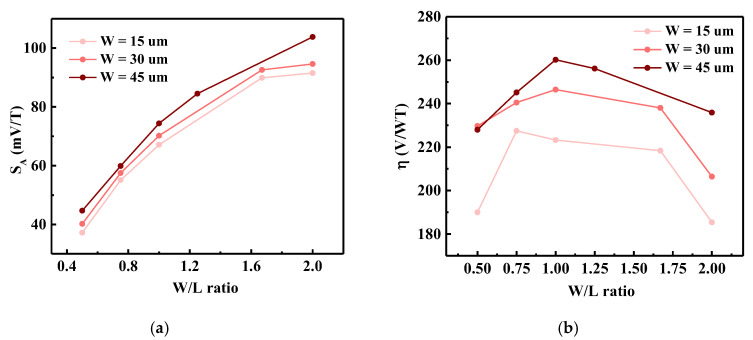
Simulated (**a**) absolute sensitivity (*S_A_*) and (**b**) efficiency factor (*η*) as a function of the *W/L* ratio for different sensor widths.

It is commonly known that the on-state drain-source current is proportional to the aspect ratio of a MOSFET. This means that the bias current of an FD-SOI Hall sensor is also determined by the *W/L* aspect ratio, which also affects the value of *S_A_*. Simulations were conducted for three different sensor widths, namely 15 µm, 30 µm, and 45 µm, and the results are presented in Figure 6a. It can be seen that as the *W/L* ratio increases, the value of *S_A_* increases accordingly. Moreover, for the same aspect ratio, the larger the width, the larger the value of *S_A_*.

However, a larger *W/L* ratio of the sensor is not always beneficial. As can be seen from Figure 6b, as the *W/L* ratio increases, the efficiency *η* of the sensor first increases and then decreases, reaching the maximum when *W/L* = 1. The *η* factor defines how much *V_H_* is generated when 1 W is consumed at 1 T of magnetic induction [24]:

(10) $$\eta_{\;}=\frac{V_{H}}{V_{bias}{\cdot I}_{bias\;}\cdot B}\;[\frac{V}{W\cdot T}].$$

Thus, it can be concluded that high-symmetry square sensors exhibit the best power efficiency, which is the best trade-off between power consumption and current-related sensitivity.

### 3.6. Contact Length/Width

**Figure 7:**
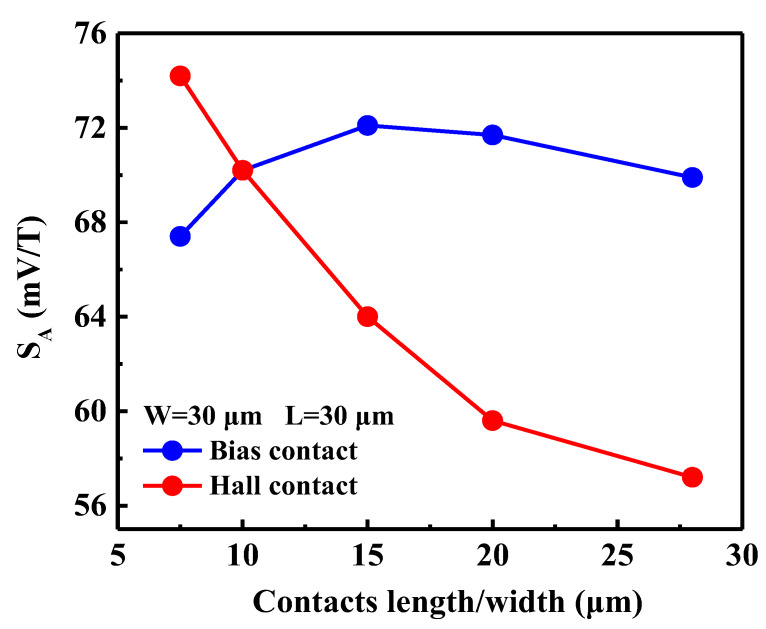
Simulations on a 30 µm × 30 µm square sensor with different lengths *L_a_* of Hall contacts and widths *W_b_* of bias contacts.

Figure 7 presents the simulated *S_A_* versus the contact length/width. The value of *S_A_* was found to increase with the decrease of the length of the Hall contacts. This is because the smaller the length of the Hall contacts, the larger the geometrical correction factor *G* (the maximum value tends to be 1) [25]. Therefore, the smaller the length of the Hall contacts, the more accurate the measurement, and the more optimal the sensitivity. However, if the length of the Hall contacts is too small and affects the overall symmetry of the sensor, the value of *V_offset_* will increase instead. The simulation proves that the value of *V_offset_* decreases with the increase of the length.

On the other hand, with the increase of the width of bias contacts, the value of *S_A_* first increases and reaches its peak at *W* = 15 μm before starting to decrease. Increasing the width of the bias contacts is similar to increasing the channel width of a MOSFET; thus, the value of *I_bias_* will increase, and the value *S_A_* will increase initially. However, as the width continues to increase, when the sensor is operating, the carrier concentration at the channel near the bias contacts increases, resulting in an increase in the overall carrier concentration *n*. The change of *n* dominates in the later period of increasing the width, resulting in a decrease in *S_A_*. In summary, for the improved sensitivity of the sensor, it is optimal to select the length of the Hall contacts as 0.25 × *L* and the width of the bias contacts as 0.5 × *W*.

### 3.7. Voltage Regulation

**Figure 8:**
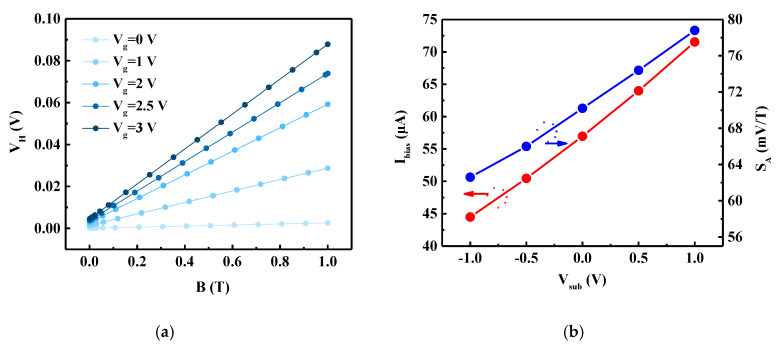
(**a**) Hall voltage (*V_H_*) versus magnetic induction (*B*) simulated for sensors with different gate voltages (*V_g_*); (**b**) Bias current (*I_bias_*) and absolute sensitivity (*S_A_*) versus substrate voltage (*V_sub_*).

One of the advantages of this FD-SOI Hall sensor is that it can regulate the bias current via the regulation of *V_g_* and *V_sub_* to achieve optimal sensitivity. As described in Section 3.1, *V_g_* can regulate *I_bias_*. The *V_H_* value of the sensor under the same magnetic induction and different gate voltages is presented in Figure 8a. When *V_g_* = 0 V, the value of *V_H_* is minimal, and with the increase of *V_g_*, *V_H_* gradually increases. This demonstrates that the gate can control whether or not the sensor will work. To avoid the increase of the gate leakage current and gate oxide breakdown, the value of *V_g_* cannot be too large [26].

In the SOI structure, the substrate (also called the back gate) can also regulate the potential of the channel in the silicon film, which promotes the ability of the gate to control the channel and increases the current drivability of the device. As can be seen from Figure 8b, with the change of *V_sub_* from −1 V to 1 V, *I_bias_* increases from 44.5 µA to 71.53 µA. Meanwhile, *S_A_* increases from 62.6 mV/T to 78.8 mV/T, an increase of 25.88%. This demonstrates that properly increasing the substrate bias voltage can increase the sensitivity of the sensor.

### 3.8. Temperature Characteristics

**Figure 9:**
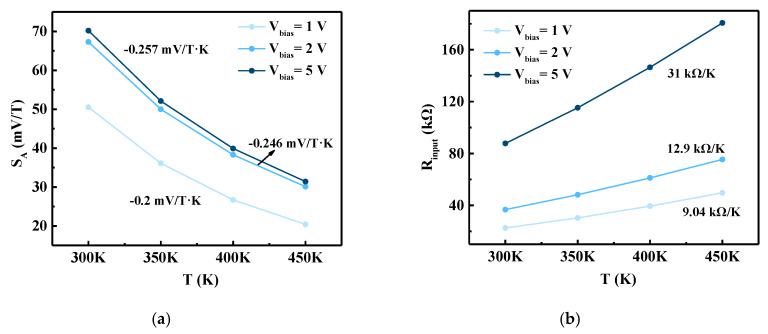
Simulated (**a**) absolute sensitivity (*S_A_*) and (**b**) input resistance (*R_input_*) as a function of temperature (*T*) with different bias voltages (*V_bias_*). The numbers in the figure are the slopes of linear fitting.

The effect of temperature on the FD-SOI Hall sensor behavior was studied by changing the ambient temperature from 300 K to 450 K. Figure 9a presents the value of *S_A_* versus the temperature at different bias voltages. As the temperature increases, the lattice vibration of the sensor materials gradually increases during operation, resulting in a decrease of carrier mobility; this reduces the *I_bias_* value of the sensor and ultimately affects the absolute sensitivity. Via linear fitting, the linear temperature drift of *S_A_* at *V_bias_* = 1, 2, and 5 V was determined to be −0.2, −0.246, and −0.257 mV/T·K, respectively.

Furthermore, the input resistance (*R_input_*) of the sensor was calculated against the temperature, as presented in Figure 9b. As the temperature increases, the *I_bias_* value increases, resulting in a decrease in the value of *R_input_*. Linear fitting was added to the variation, and the linear temperature drift of *R_input_* at *V_bias_* = 1, 2, and 5 V was determined to be 9.04, 12.9, and 31 kΩ/K, respectively.

### 3.9. Summary and Discussion

When Hall sensor technology is selected, performance implementation (such as high sensitivity, low offset, and high efficiency) and bias conditions are important factors to consider. One of the factors that affects performance is the structural parameters of the sensor. A nearly-square or high-symmetry structure has not only proper absolute sensitivity, but also maximum efficiency (Figure 6). Moreover, the length of the contacts affects the sensitivity and offset voltage, and the Hall contacts length of 0.25 × *L* and the bias contacts width of 0.5 × *W* are the optimal choices (Figure 7). Another way to improve sensor performance is to choose an appropriate gate oxide thickness and gate work function; the gate oxide thickness should be around 100 nm, while the gate work function should be relatively small.

Furthermore, bias conditions are other factors that must be considered. In addition to conventional bias voltage, gate and substrate regulation were also analyzed in this work. The bias voltage is prone to a sensitivity/offset trade-off (Figure 3), so the choice of the optimum value for the bias current is driven by the target application. The gate voltage can control the switch of the sensor; thus, the addition of a clock signal with a signal processing circuit can be applied in Hall integrated circuits. The introduction of substrate regulation increases the diversity and integrity of the bias, and properly increasing the substrate voltage can improve the performance of the sensor (Figure 8).

Table 1 summarizes the simulation results of the performance of the Hall sensors, and presents a direct comparison of the performance of Hall sensors in bulk versus FD-SOI technology. The results of the FD-SOI structure indicate its high sensitivity and high efficiency. Not only is the absolute sensitivity slightly larger than that reported by Paun et al. [21], but the current-related sensitivity is much greater. In addition, via comparison, it can be concluded that there is an increase of more than three times in the efficiency of the FD-SOI Hall sensor. Despite the higher offset voltage of the FD-SOI architecture, it can be adjusted and improved by the bias voltage and the length of the Hall contacts.

The most important consideration is that the bias current of the FD-SOI architecture is at the microamp level, rather than at the milliamp level as in most other architectures, which leads to the low power consumption of the sensor. Moreover, to achieve the same Hall voltage, the FD-SOI architecture requires relatively small current bias. As shown in (10), at the same Hall voltage the efficiency of the sensor is inversely proportional to the bias current, so the smaller bias current of the sensor in this work leads to higher efficiency.

## 4. Conclusions

This work investigated the impacts of the aspect ratio, doping concentration, gate oxide and top silicon film thicknesses, and bias conditions on the performance of FD-SOI Hall sensors. The sensor configurations were evaluated in terms of the Hall voltage, sensitivity, efficiency, offset, and temperature characteristics via TCAD simulations based on Synopsys Sentaurus^®^. Table 2 summarizes the simulated Hall sensor parameters and the effect of parameters changes on performance.

The following are the optimal design rules and important trade-offs of FD-SOI Hall sensors that designers should consider for practical applications.

- The aspect ratio restricts the trade-off between sensitivity and efficiency, and a square structure can be optimal for the balance between sensitivity and efficiency.
- To achieve the best sensitivity, the length of the Hall contacts and the width of the bias contacts are suggested to be 0.25 × *L* and 0.5 × *W*, respectively.
- From the perspective of sensor performance optimization, materials with a small work function should be selected for the gate, and the most suitable gate oxide thickness is 100 nm.
- The bias voltage affects not only the sensitivity, but also the offset voltage. It should be selected according to real applications.
- Properly increasing the substrate voltage can improve sensitivity. The gate can control whether the device is turned on or off, and the sensor can therefore be better applied in Hall integrated circuits.

These design rules can guide designers to accurately model and characterize FD-SOI Hall sensors. They can choose the appropriate parameters and biases based on the priority of the figures of merit in the circuit.

## Figures and Tables

**Table 1:** Simulated Hall sensor performance summary and comparison.

	Architecture	Active Area Size (μm)	Bias	*S_A_* (mV/T)	*η* (V/WT)	*V_offset_* (mV)
[21]	Bulk	54 × 54	*I_bias_* = 1 mA	77.83	41.48	- ^1^
[24]	Bulk	30 × 30	*I_bias_* = 1 mA	0.25	75	-
[13]	PD-SOI	50 × 50	*I_bias_* = 1 mA	55	-	0.025
This work	FD-SOI	30 × 30	*V_bias_* = 5 V (*I_bias_* = 56 μA)	86.5	218.9	3.04

^1^ - indicates that it has not been analyzed in corresponding work.

**Table 2:** Impacts of simulated Hall sensor parameters changes on performance ^1^.

Parameters	Values	Effect on Performance			
		Change	*S_A_*	*η*	*V_offset_*
W/L	30 μm/30 μm	↑	↑	↗↘ ^2^	-
Hall contacts length	0.25 × *L*	↑	↓	-	↓
Bias contacts width	0.5 × *W*	↑	↗↘	-	-
Doping concentration	1 × 10^16^ cm^−3^	↑	↓	-	-
Silicon film thicknesses	50 nm	↑	↓	-	-
Gate oxide thickness	100 nm	↑	↓	-	-
Bias voltage	5 V	↑	↑	-	↑
Substrate voltage	1 V	↑	↑	-	-
Gate voltage	2.5 V	↑	↑	-	-

^1^ ↑ and ↓ indicate an increase or decrease in value while - indicates that it has not been analyzed in this work. ^2^ ↗↘ indicates that the value increases first and then decreases.
